# The impact of re‐characterizing metastatic pancreatic neuroendocrine tumors: A prospective study

**DOI:** 10.1111/jne.70040

**Published:** 2025-05-05

**Authors:** Kazhan Mollazadegan, Johan Botling, Britt Skogseid, Barbro Eriksson, Lovisa Falkman, Liang Zhang, Ieva Lase, Staffan Welin, Anders Sundin, Joakim Crona

**Affiliations:** ^1^ Department of Medical Sciences Uppsala University Uppsala Sweden; ^2^ Department of Immunology, Genetics and Pathology Uppsala University Uppsala Sweden; ^3^ Department of Surgical Sciences Uppsala University Uppsala Sweden

**Keywords:** hormones, Ki‐67, pancreatic neuroendocrine tumor, PET/CT, re‐characterization

## Abstract

The biology of metastatic pancreatic neuroendocrine tumors (panNET) may alter over time. It remains to be defined if, how, and when this patient group should be recommended to re‐evaluate the characteristics of their disease. This prospective single‐center, longitudinal cohort study at Uppsala University Hospital, Sweden (NCT03130205), included metastatic panNET patients with progressive disease to participate in a standardized re‐characterization protocol: clinical and biochemical analyses, core‐needle biopsy, and dual‐positron emission tomography/computed tomography (PET/CT) (^18^F‐fluorodeoxyglucose (^18^F‐FDG) and Gallium‐68 DOTATOC (^68^Ga‐DOTATOC)) with NETPET score assessments. At further disease progression, a second re‐characterization was offered. The proportion of patients with a clinically significant change is reported and defined as information that could lead to a change in the therapeutic algorithm proposed in the European Neuroendocrine Tumor Society (ENETS) guidelines. Between 2017 and 2021, 21 patients with progressive metastatic panNETs were included. Before inclusion, 19 tumors were grade (G) 1 or 2, and two were G3. Sixteen patients underwent biopsy with collection of adequate tumor material, of whom 81.3% (*n* = 13/16) displayed an increase in the Ki‐67 index, with transition from G2 to G3 in 50% (*n* = 8/16). Twelve and 15 patients were positive on ^18^F‐FDG‐ and ^68^Ga‐DOTATOC‐positron emission tomography (PET), respectively. This corresponded to NETPET grades P1 (*n* = 2), P2b (*n* = 12), and P3b (*n* = 1). A clinically significant change was noted among 62% (*n* = 13/21) of patients at first re‐characterization, leading to therapy change in 7 positron emission tomography/computed tomography (PET/CT) patients. After the second re‐characterization, a significant clinical change occurred in 43% (*n* = 3/7) with a shift in therapy for one patient. This study shows that a considerable number of progressive metastatic panNETs experience significant changes in their disease characteristics over time. This may result in a revised treatment plan and highlights the need to re‐evaluate all relevant aspects of panNET disease. Such comprehensive re‐characterization is particularly crucial in the context of clinical trial inclusion.

## 1 INTRODUCTION

Metastatic pancreatic neuroendocrine tumors (panNET) currently lack a definitive cure, and early‐stage surgical intervention is believed to represent the only treatment with curative potential.^1^, ^2^, ^3^, ^4^ PanNETs exhibit a broad spectrum of disease characteristics, ranging from indolent to rapidly growing tumors, often accompanied by the secretion of various hormones. Together, these factors significantly affect the prognosis in disseminated disease and form the basis for the therapeutic algorithm.^5^, ^6^ PanNETs also display spatial and temporal heterogeneity^5^ and may transition from low/intermediate grade to high‐grade disease. This phenomenon has also been observed in NETs of lung origin.^7^, ^8^ Increases in panNET grade may be accompanied by alterations in hormone secretion patterns.^6^, ^9^ Metachronous progression from a low/intermediate grade to a high‐grade panNET (secondary panNET G3) is a concerning development, as it has been associated with very poor outcomes.^10^ Consequently, the management of metastatic panNETs poses a significant challenge.^11^

To comprehensively understand the individual nature of each panNET patient's disease, multiple diagnostic investigations are employed to form subsequent treatment strategies and follow‐up protocols. These investigations encompass the evaluation of hormones and symptoms (hormonal syndromes), determination of tumor differentiation and proliferation (tumor grade), and utilizing positron emission tomography/computed tomography (PET/CT) with gallium(Ga)‐68 labeled somatostatin analogs (SSA), such as ^68^Ga‐DOTATOC and [^18^F]fluorodeoxyglucose (FDG) to assess tumor stage, as well as somatostatin receptor (SSTR) status and metabolic activity. The utilization of ^18^F‐FDG‐PET, either alone or in conjunction with ^68^Ga‐DOTA‐SSA‐PET, has exhibited potential in predicting prognosis, response to specific systemic therapies, and tumor grade in well‐differentiated gastroenteropancreatic NET.^12^, ^13^ The NETPET scoring system^14^ was developed to be used as a single parameter for prognosis by integrating the results from dual‐PET with ^68^Ga‐DOTA‐SSA and ^18^F‐FDG in patients with metastatic NET. It was shown to provide better information on prognosis compared to tumor grade.

The current understanding of NET disease progression is primarily derived from retrospective studies, typically focusing on how a single parameter (e.g., Ki‐67 or hormones) changes over time. We, therefore, aimed to prospectively investigate the impact of re‐characterization in metastatic panNET at the point of disease progression, examining changes in all relevant parameters: histopathology, dual PET‐imaging, and hormone secretion profile. We hypothesized that a substantial proportion of patients experience clinically significant changes in their disease characteristics, impacting their management and clinical outcomes.

## 2 METHODS

This study received approval from the Ethical Review Board in Uppsala (reference ID: 2016/277), and written informed consent was obtained from all patients. The reporting of this study was based on the guidelines outlined in the Strengthening the Reporting of Observational Studies in Epidemiology (STROBE) guidelines, with relevant adaptations to fit our unique dataset.^15^ Follow‐up data collection concluded on August 30, 2023.

### 2.1 Study design, setting, and participants

This was a single‐center, longitudinal cohort study analyzing patients enrolled in a prospective protocol for panNET disease re‐characterization at Uppsala University Hospital. This protocol included patients that fulfilled the following criteria: (1) age ≥18 years, (2) informed consent, (3) pathology‐confirmed diagnosis of panNET (World Health Organization [WHO] 2017) grade(G) G1–G3, (4) inoperable, stage III or IV (according to the 8th TNM classification edition for NET), (5) WHO performance status ≤2, (6) progressive disease (as defined by the responsible physician), and (7) biopsy procedure not associated with inappropriate risk. Progressive disease was defined as progression based on clinical, radiological, and biochemical parameters, as assessed by the treating physician and the study coordinator (JC). Radiological progression was the primary and most consistent inclusion criterion. While biochemical and clinical indicators contributed to the assessment, they were not used in isolation for patient selection but rather in conjunction with imaging findings.

**FIGURE 1:**
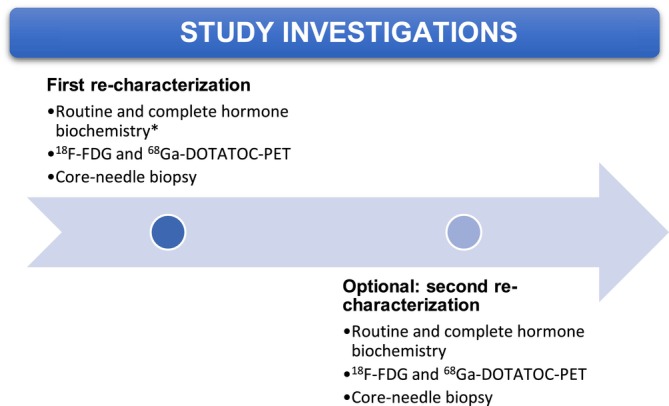
Re‐characterization protocol. Patients with a pathology‐confirmed diagnosis of metastasized panNET (WHO 2017) G1‐G3 were included at disease progression and underwent re‐characterization. *Adrenocorticotropic hormone (ACTH), gastrin, pancreatic polypeptide (PP), calcitonin, insulin, proinsulin, C‐peptide, vasoactive intestinal peptide (VIP), glucagon, chromogranin A. In selected cases, serum/urine 5‐hydroxyindoleacetic acid and parathyroid hormone‐related peptide (PTH‐rp) were collected.

Initially, a first re‐characterization was performed upon study entry for each patient (as outlined in Figure 1). We collected the following data and biomaterials: (1) core‐needle biopsy, (2) PET/CT with ^18^F‐FDG and ^68^Ga‐DOTATOC‐PET, including diagnostic intravenous contrast‐enhanced CT, as well as (3) a comprehensive panel of pancreatic hormone analyses. If any signs of disease progression were observed after inclusion, an optional second re‐characterization was offered.

### 2.2 Study objectives, variables, and method for outcome reporting

Data analyses were finalized once follow‐up for all patients was complete. Our objective was to describe the results and clinical impact of re‐characterizing metastasized panNET patients. First, we aimed to investigate the proportion of patients exhibiting clinically significant changes in disease characteristics, defined as (i) the occurrence of a new hormonal syndrome or relevant hormone secretion, (ii) changes in PET‐tracer uptake pattern (loss or gain of ^18^F‐FDG‐ or ^68^Ga‐DOTATOC‐positive tumor lesions), (iii) an increase in Ki‐67 index resulting in a transition from low grade to a higher grade panNET, or from low G2 (Ki‐67 3% to <10%) to high G2 (Ki‐67 >10% to <20%), leading to changes in treatment strategy according to European Neuroendocrine Tumor Society (ENETS) guidelines.^1^, ^2^ Secondly, we aimed to describe disease evolution of panNETs in an unselected cohort with progressive disease by using the collected data and biomaterials. Finally, we sought to explore the potential use of functional imaging as a minimally invasive method to detect increases in tumor proliferation.

### 2.3 Biochemistry, tumor markers, and hormones

A comprehensive panel of relevant hormone and neuropeptide analyses was conducted according to established clinical routines during re‐characterization, and hormonal syndromes were classified accordingly. The hormones analyzed were predetermined in the study protocol and were collected for all patients at study inclusion and further progression. These hormones and neuropeptides included insulin, pro‐insulin, c‐peptide, glucagon, calcitonin, gastrin, pancreatic polypeptide (PP), vasoactive intestinal peptide (VIP), and adrenocorticotropic hormone (ACTH). The measurement of urinary or serum 5‐hydroxyindoleacetic acid (5‐HIAA) was limited to cases where symptoms suggestive of the carcinoid syndrome were observed. Additionally, patients with hypercalcemia underwent evaluation of parathyroid hormone‐related peptide (PTHrp) levels. Only elevated hormones related to the tumor disease were measured in the intermediate period. The acquired data were validated to comply with ENETS guidelines.^2^, ^16^

### 2.4 Core‐needle biopsy and histopathology

All patients were scheduled for trans‐abdominal ultrasound‐guided biopsy, according to standard procedures, using an 18‐gauge core needle. Progressive tumor lesions or new metastases were selected, primarily favoring previously SSTR‐positive lesions turning FDG‐avid for biopsy. Tumor tissue staining and immunohistochemical analyses were performed according to clinical routine based on the ENETS guidelines.^16^ Staging was determined by TNM Classification of Malignant Tumors, 8th edition (2016), with grading and classification according to the 4th edition WHO Classification of Tumors of Endocrine Organs, published in 2017.^17^ The safety of core‐needle biopsy was described retrospectively according to Common Terminology Criteria for Adverse Events (CTCAE) version 5.0.^18^

### 2.5 Molecular imaging

Dual‐PET/CT with ^68^Ga‐DOTATOC and ^18^F‐FDG were re‐analyzed by using the NETPET scoring system, which uses the tumor's negativity or degree of positivity (uptake) of ^68^Ga‐DOTATOC and ^18^F‐FDG, respectively, to generate a combined score.^14^ Each pair of PET examinations was independently reviewed and analyzed by a consultant radiologist with 30 years of PET experience (AS) and a resident physician (KM), respectively. When there was a disparity in NETPET scores between the readers, these examinations were re‐evaluated and scored by the readers in consensus. PET scoring was performed by reviewing the ^68^Ga‐DOTATOC and ^18^F‐FDG scans displayed in the transverse and coronal imaging planes. Additionally, rotating maximum intensity projection images were utilized, which were anatomically aligned and synchronized to ensure coordinated movement and scrolling of the images. The PET scans were displayed by applying standardized uptake value (SUV) pre‐sets, with SUV range 0–7 for ^18^F‐FDG‐PET and SUV range 0–15 for ^68^Ga‐DOTATOC‐PET. The lesions exhibiting the most significant disparity between ^18^FDG avidity and ^68^Ga‐DOTATOC uptake were identified. The scans were then graded based on the NETPET‐score system by using the following criteria: P0: SSTR and FDG negative, P1: SSTR positive, FDG negative, P2a: FDG<SSTR (1–2 lesions), P2b: FDG<SSTR (3 or more lesions), P3a: FDG = SSTR (1–2 lesions), P3b: FDG = SSTR (3 or more lesions), P4a: FDG>SSTR (1–2 lesions), P4b: FDG>SSTR (3 or more lesions), P5: FDG positive, SSTR negative.

## 3 RESULTS

Between 2017 and 2021, 21 patients (male *n* = 11, female *n* = 10) with progressive metastatic panNET were included for disease re‐characterization. The median time from primary diagnosis to study inclusion was 35 months (range: 2–181 months). Eighteen patients had radiological progression while on systemic therapies. Two patients showed distinct clinical and radiological progression shortly after their initial diagnosis, which was suggestive of aggressive disease characteristics. At primary diagnosis, all patients had stage III (*n* = 1) or IV (*n* = 20) disease, median Ki‐67 was 10% (range: 2%–31%), and 19 patients harbored G1 or G2 tumors, while two had G3 tumors. There was one patient with multiple endocrine neoplasia type 1 (MEN1) syndrome. Before study inclusion, there were five patients with functioning panNETs and 16 with non‐functioning disease. Patients had received a median of 2 (range 1–4) treatment lines. Conventional systemic therapies given in the cohort prior to inclusion were alkylating chemotherapy (*n* = 15), peptide receptor radionuclide therapy (PRRT) (*n* = 8), everolimus (*n* = 3), and somatostatin analogs (*n* = 12) (Table 1).

**TABLE 1:** Patients' characteristics at inclusion.

Demographics	
Patients included, *n*	21
Age, median (range)	65 (35–80)
Gender, male/female	11/10
Sporadic disease, *n*	20
MEN1 syndrome, *n*	1
Tumor characteristics at diagnosis	
Stage (UICC 8th edition), *n*	
I	0
II	0
III	1
IV	20
Biopsy localization, *n*	
Liver metastasis	19
Primary tumor (primary surgery)	2
Grade (WHO 2017), *n*	
G1	1
G2	18
G3	2
Ki‐67 index, median (range)	10% (2–31)
Hormonal syndrome, *n*	2 (+ 1 with PTHrp)
Hormone secretion, *n*	5
Treatment lines before first re‐characterization, *n*	
0	3
1	4
2	6
3 or more	7
Previous therapies	
No previous systemic therapy	3
Previous systemic therapy	18
Alkylating chemotherapy	15
PRRT	8
mTORi	3
Locoregional therapy	4
Primary surgery	2
Re‐operated	1
SSA	12

Abbreviations: alkylating chemotherapy: temozolomide, streptozotocin; locoregional therapy: radiofrequency ablation, external radiotherapy, resection of liver metastasis; MEN1: multiple endocrine neoplasia type 1; mTORi, Mammalian Target of Rapamycin inhibitor (everolimus); PRRT, peptide receptor radionuclide therapy; SSA, somatostatin analogs.

### 3.1 Results of first re‐characterization

**FIGURE 2:**
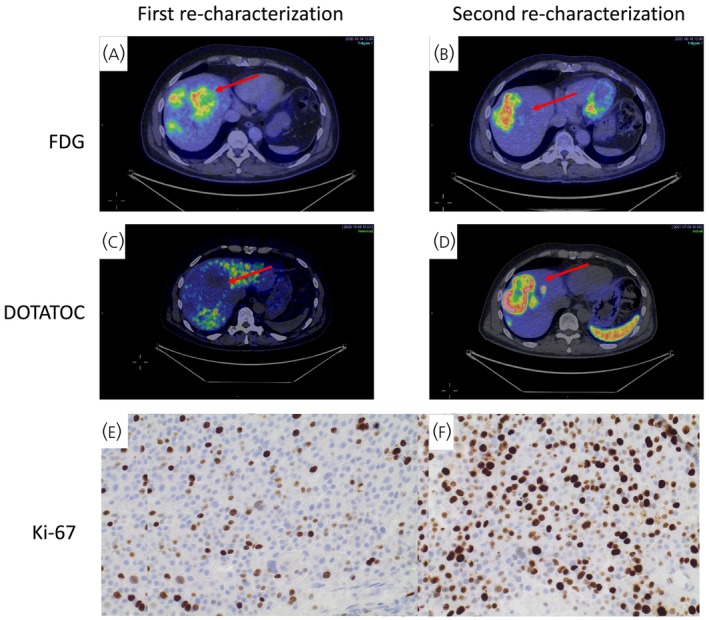
(A–F) A patient with liver metastasized panNET G2 was included at disease progression and had the following results after first re‐characterization: (A). ^18^F‐FDG‐PET showed positive uptake of liver metastases, (C) while the same lesions were negative on ^68^Ga‐DOTATOC‐PET, (E) immunohistochemical staining of the core‐needle biopsy from liver metastasis revealed NET G2 with Ki‐67 18%. A second re‐characterization was performed 1 year later due to disease progression: (B). ^18^F‐FDG‐PET scan revealed a new hypermetabolic lesion in the liver, alongside a decrease in size and uptake of other previously identified FDG‐positive lesions—meanwhile, the (D). ^68^Ga‐DOTATOC‐PET, which was previously negative, demonstrated positive uptake in the known metastatic ^18^F‐FDG positive lesions and the newly identified liver lesion detected by ^18^F‐FDG‐PET. Additionally, (F) immunohistochemical staining of new core‐needle liver biopsy revealed an increase in Ki‐67 to 40% and a transition in grade to NET G3.

Fifteen of 21 patients underwent dual‐PET/CT scans, and 16/21 underwent successful core‐needle biopsy (yielding representative tumor tissue that was evaluable for histopathological re‐characterization). Thirteen out of 21 patients (62%) experienced a significant clinical change at the first re‐characterization (Table 2 and Table S1). Amongst them, four patients exhibited secretion of new hormones and neuropeptides (calcitonin, gastrin, VIP, PP), but without developing a hormonal syndrome. Histopathological analysis was unavailable for five patients due to unsuccessful tissue acquisition (no representative tumor tissue was obtained). Among them, two patients harbored lesions that were undetectable by ultrasound, and subsequent CT‐guided core‐needle biopsies failed to yield representative tumor tissue. Of the 16 patients with successful histopathological re‐characterization, the median Ki‐67 was 16% (range 6–70), with seven G2 and nine G3 tumors. There was a transition from G2 to G3 in eight patients (Figure 2; a case with G2 to G3 progression) and from low to high G2 in three additional cases (Table 2; for more details regarding progression‐related changes, see Table S1).

**TABLE 2:** Summary of results from first and second re‐characterization.

	1st re‐characterization	2nd re‐characterization
Significant clinical change yes/no	*n* = 13/21	*n* = 3/7
Therapy change yes/no	*n* = 7/13	n = 1/3
Histopathology		
Biopsy localization		
Liver metastasis (new or progressive)	*n* = 21/21	*n* = 4
FDG avid lesion	*n* = 8/21	n/a
Surgery		
Liver resection (metastasis)	*n* = 0	*n* = 1
Grade (WHO 2017)		
G1	*n* = 0	*n* = 0
G2	*n* = 7	*n* = 3
G3	*n* = 9	*n* = 2
Median Ki‐67 (%) (range)	16 (6–70)	14 (10–33)
Comparison to previous results		
Increase in Ki‐67 index yes/no/NA	*n* = 13/3/5	*n* = 4/0/3
G2 to G3	*n* = 8	*n* = 1
Low G2 to high G2	*n* = 3	*n* = 1
Functional imaging		
FDG‐PET+/−/NA	*n* = 17/4/0	*n* = 3/1/3
Median SUV_max_ (range)	9 (6–20)	13 (9–13)
SSTR‐PET+/−/NA	*n* = 15/0/6	*n* = 4/1/2
Hormone analysis		
New hormone secretion yes/no	*n* = 4/17	*n* = 2/5
New hormonal syndrome yes/no	*n* = 0/21	*n* = 0/7

*Note*: Significant clinical change definition: the occurrence of a new hormonal syndrome or relevant hormone secretion, changes in PET‐tracer uptake pattern (loss or gain of ^18^F‐FDG‐ or ^68^Ga‐DOTATOC‐positive tumor lesions), an increase in Ki‐67 index resulting in a transition from a low grade to a higher grade panNET, or from low G2 to high G2 panNET, leading to changes in treatment strategy according to ENETs guidelines^1^, ^2^; NA equals unsuccessful biopsy: tumor lesions not visualizable or not accessible or material not containing representative tumor tissue; low G2 to high G2: low G2 Ki‐67 <10%, high G2 Ki‐67 >10%, cut off values for Ki‐67 retrieved from European Neuroendocrine Tumor Society (ENETS) Consensus Guidelines, 2016.

The safety of core‐needle biopsy was retrospectively assessed following CTCAE 5.0 criteria. One patient experienced a grade 3 adverse event, which included hepatic hemorrhage, anemia, and abdominal pain.

Fifteen patients underwent dual‐PET investigation; 13 had tumor uptake of both ^68^Ga‐DOTATOC and ^18^F‐FDG. Six patients had only undergone ^18^FDG‐PET/CT, of whom four harbored ^18^F‐FDG‐positive tumors, and two had ^18^F‐FDG‐negative tumors. Among the 15 patients who underwent dual‐PET/CT, 12 were classified as P2b according to the NETPET grading score, while two patients were classified as P1 and one patient as P3b (Table S2).

Seven patients' treatment plans were modified based on the new histopathological, biochemical, and functional imaging findings following re‐characterization.

### 3.2 Results of second re‐characterization

Of all eligible patients included in the study, only 7 patients progressed during the study period and underwent a second re‐characterization, and a clinically significant change was observed in 43% (*n* = 3), leading to therapy changes in one patient (Table S4). Two patients had new hormone secretions (VIP, gastrin) but did not develop a hormonal syndrome. Five patients underwent new histopathological analyses: four had core‐needle biopsies, and one underwent liver surgery for a progressive liver lesion. Among these patients, four exhibited an increase in the Ki‐67 index relative to their first re‐characterization, with one progressing from G2 to G3 and another from low G2 to high G2 (see Table 2 and Table S4). Two patients demonstrated a twofold increase in their Ki‐67 index compared to that at their first re‐characterization. The median Ki‐67 index was 14% (range: 10–33). All seven patients underwent PET/CT, dual‐PET/CT (*n* = 2), ^68^Ga‐DOTATOC‐PET/CT (*n* = *3*), and ^18^F‐FDG‐PET/CT (*n* = *2*). Three showed FDG‐avid tumors, and four were ^68^Ga‐DOTATOC‐positive. One patient who was FDG‐negative at first re‐characterization had a new FDG‐avid lesion. Within the subset of patients who underwent dual‐PET/CT, one was scored as P2b and the other as P1 (Table S5).

### 3.3 Patient outcomes

Seven patients were alive at the last follow‐up. Overall survival (OS) was measured from the time of study inclusion, and the median survival was 20 months (range 2–36 months, Table S3). All patients with NETPET score P1, and 6 out of 12 P2 patients, were still alive at the last study follow‐up. Four patients died shortly after being included in the study (for details, refer to Table S3).

## 4 DISCUSSION

This is the first prospective study on how panNET biology changes over time across all clinically relevant dimensions: hormone spectrum, tumor cell proliferation, and functional imaging profile.

At the first disease re‐characterization, nearly two‐thirds (62%, 13/21) of patients showed changes that resulted in modified patient management in 7/13. Additionally, three out of seven patients experienced further clinically relevant changes during their second re‐characterization. These findings highlight the potential significance of re‐evaluating the biology of metastatic panNET, particularly at the time of disease progression.

### 4.1 Interpretation

In this research project, we aimed to generate data to determine if, when, and how disease re‐characterization of metastatic panNET should be performed. The most significant finding was that most patients experienced clinically relevant changes in disease biology, which led to alterations in their management. This underscores the importance of re‐evaluating panNET biology, which could lead to improved treatment strategies. The timepoint for such reassessment still needs to be better defined. Most patients (*n* = 18) were included at disease progression on systemic therapies, which is a logical time for re‐evaluating the management plan. Due to the heterogeneous nature of this disease, we propose that disease re‐characterization should involve all relevant dimensions: symptoms, hormones, tumor proliferation, and functional imaging profiles. An individualized approach is needed in the clinical setting to balance different interventions' potential benefits and risks. In the research setting, we argue for the benefit of a comprehensive re‐characterization in the case of clinical trial inclusion.

**FIGURE 3:**
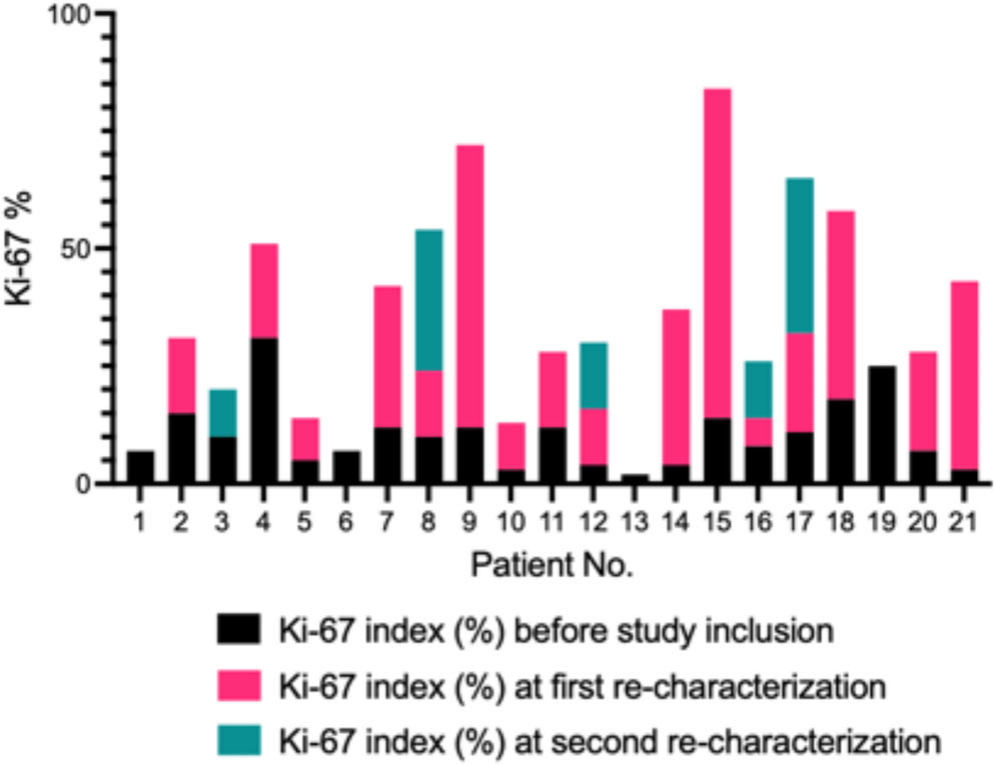
Ki‐67 changes observed after re‐characterization. Patient No. 3 underwent core‐needle biopsy at first re‐characterization, but tumor tissue was not detectable. Therefore, only values from the second re‐characterization are available and presented.

We also used the collected data to draw a general figure on how the biology of metastatic panNET changes during the disease course: changes towards more aggressive tumor biology could be identified in most cases (Figure 3). This data builds on previous evidence and demonstrates that metastatic panNET is a very dynamic condition. Consistent with previous studies, a subset of PanNET patients undergo significant tumor evolution over time. Zhang et al. reported Ki‐67 variations in nearly half (48.5%) of cases, with 17.5% experiencing a grade increase. Metachronous metastases showed greater Ki‐67 variation, and high‐grade metastases were linked to shorter progression‐free survival and OS.^6^ In another recent study, 24.2% of initially low‐grade G1 or G2 panNET progressed to high‐grade (G3), median Ki‐67 increase of 27%, with poorer overall survival, and progression was more common in heavily pretreated patients.^19^ Similarly, Bourdeleau et al. found that 36% of cases exhibited a grade shift, and a Ki‐67 increase of ≥2% per year was independently associated with worse OS. Treatment also appeared to impact Ki‐67 progression, as patients receiving alkylating chemotherapy were more likely to experience significant Ki‐67 increases. The authors also suggest that re‐characterization should be performed at the time of progressive disease.^20^ Supporting this, Backman et al. demonstrated that exposure to alkylating agents led to an increase in tumor mutational burden (TMB) in metastatic samples compared to primary tumors. In a validation cohort of 24 patients, 33% developed high TMB (≥50) following alkylating chemotherapy, with most cases exhibiting mismatch repair (MMR) mutations and progressing to high‐grade tumors.^21^ These findings suggest a potential link between alkylating therapy, hypermutation, and tumor grade progression, further emphasizing the importance of re‐characterization, particularly at disease progression.

PanNETs have been documented to exhibit multiple and secondary hormone secretion in 9.3% of patients, with increased morbidity and mortality.^22^ Metachronous hormone secretion is described as a rare phenomenon and is linked to more aggressive and poorer outcomes. In a retrospective multicenter study by de Mestier L. et al.,^23^ metachronous hormone syndromes were found in 3,4% of 435 patients with panNET and were linked to panNET progression and increased Ki‐67 indices; OS was described to be 28 months (range: 3–56). While we failed to conclusively determine the sole impact of hormone secretion on morbidity due to the limited study population, we still observed the advent of hormonal secretion and transition to a higher tumor grade in two patients after the first re‐characterization (Table S1). In another patient, hormonal secretion developed along the course of the disease, but the tumor grade could not be determined at re‐characterization since histopathology data were unavailable. New hormone secretion was detected for a fourth patient, but there was no change in tumor grade. Although the value of measuring PP remains debated, in the case of non‐functioning panNETs, it has been previously suggested to be monitored as a tumor marker if elevated. Similarly for VIP, when there is no sign of hormonal syndromes. Since metachronous hormone secretion has been reported in previous studies, we could not exclude any potential hormone or neuropeptide that might serve as a prognostic indicator. Further analysis is needed to determine the benefit of screening for all hormones at the time of progression in a larger population.

We also aimed to use this dataset to evaluate whether minimally invasive procedures could capture relevant changes in tumor proliferation. Because of the complex and evolving nature of panNETs, some centers advocate for repeated tumor biopsies to better understand tumor biology and to allow for detecting changes during the disease course. However, while such re‐characterization may provide refined prognostic markers, the invasive nature of tumor biopsies limits their clinical utility. It is susceptible to sampling errors caused by heterogeneity within and between lesions. Although generally a safe procedure, repeated biopsies may not be appropriate for all patients with metastatic panNET. Even when tumor progression or dedifferentiation is suspected, the decision to perform biopsies must be made with careful consideration. These difficulties are illustrated by the fact that seven patients (first re‐characterization, *n* = 6, second re‐characterization, *n* = 1) in our study at re‐biopsy exhibited minor changes in tumor Ki‐67 index that did not warrant a change of therapy. Biopsy in two patients resulted in a lower Ki‐67 index than at primary diagnosis (Table S1), reflecting intra‐ and intertumoral heterogeneity of metastatic lesions in panNETs.^20^, ^24^

The potential prognostic utility of ^18^F‐FDG‐PET imaging has been explored in previous studies, which have shown shorter OS in patients with ^18^F‐FDG‐positive tumors.^12^, ^25^ The utilization of dual‐PET/CT imaging has, in previous research, been described to capture evolving biological changes, but our study failed to differentiate between low G2, high G2, and G3 panNET, respectively. Notably, even patients with a Ki‐67 index >50% could exhibit ^68^Ga‐DOTATOC‐ and ^18^F‐FDG‐positive tumors (Table S3). A recent study in this domain demonstrated that dual‐PET/CT can effectively predict the histopathological grade of the tumor.^26^ Another study showed a high detection rate for both tracers. However, ^18^F‐FDG‐positive tumors were more likely to be classified as G2 or higher, further emphasizing the correlation between the tumor's ^18^F‐FDG avidity and Ki‐67 index, which could be valuable in differentiating G1 from G2 tumors.^27^ Because most of our patients already harbored G2 tumors at study inclusion (Table 1), we could not assess tumor ^18^F‐FDG avidity in patients progressing from G1 to G2 tumors. ^18^F‐FDG avidity may, therefore, not always provide insight into the histopathological grade, even though it is generally considered a negative prognostic factor^12^, ^13^ (due to the higher metabolic rate of the tumors). Interpreting or predicting tumor grade with dual‐PET/CT can be challenging, as shown in Figure 2. Lesions that were previously negative on ^68^Ga‐DOTATOC‐PET but exhibited FDG‐avidity showed uptake on both ^68^Ga‐DOTATOC‐ and ^18^F‐FDG‐PET upon progression from G2 to G3. Developing integrated ^18^F‐FDG and ^68^Ga‐DOTA‐SSA‐PET grading systems, such as the NETPET score, may help improve prognostic assessment. In the validation study of the NETPET score, it demonstrated a significant association with OS and time to progression and a strong correlation to histological grade.^28^

### 4.2 Limitations

We acknowledge several limitations of our study. The single‐center design, relatively modest sample size, and the risk of selecting progressing patients with good performance status imply that the study patients may not fully represent the entire spectrum of metastatic panNET disease. Furthermore, the diverse array of prior treatment regimens before study inclusion may complicate the interpretation of our findings, particularly concerning the assessments of disease progression (Table S6, for treatment lines before and after study inclusion in detail).

Conducting a large prospective study on metastatic panNET evolution is challenging due to the rarity of the disease and the difficulty of obtaining a sufficiently large sample size. Since patients must meet specific criteria, such as evidence of progressive disease, before undergoing biopsy, not all can be sampled at the same time, and usually the time between primary biopsy and follow‐up can be from 6 months up to 44 months. The rarity of the disease and the logistical problems in obtaining serial biopsies, thus, explain why most studies on metastatic panNET evolution tend to be retrospective in nature^6^, ^9^, ^20^, ^24^, ^29^, ^30^ Albeit this was a hypothesis‐generating study, and the sample size, even if small, was thought to be sufficient to see if this way of collecting data is plausible for future studies performed similarly in a larger cohort.

Moreover, the considerable heterogeneity observed within panNETs, encompassing functioning and non‐functioning tumors alongside distinct tumor grades, including both sporadic and hereditary panNET, introduces various confounding factors when evaluating treatment responses and disease progression. The wide range between primary diagnosis and study inclusion (2 to 181 months) could influence disease progression dynamics and outcomes, i.e., two cases were re‐evaluated soon after the initial diagnosis and before starting systemic therapy. The re‐evaluation was prompted by disease progression and clinical signs indicating a more aggressive condition than the confirmed diagnosis had shown. Missing data is also a problem that may have resulted in a missed opportunity to gather important insights into disease behavior. Not all patients could undergo biopsy, and the procedure in some patients failed to provide sufficient tumor material for analysis, even though the same biopsy needle size was used for all biopsies. Also, whether re‐characterization biopsies were performed on the same or different tumor lesions were unfortunately not documented. By contrast, it is essential to acknowledge that this study contributes valuable longitudinal data on panNETs, which remains notably scarce in the current scientific literature for this rare disease. To our knowledge, this is one of the first studies to examine temporal changes across multiple parameters of disease progression.

## 5 CONCLUSIONS

Our findings, along with previous research, suggest that disease characteristics in many panNET patients may change over time across several parameters that may potentially affect treatment management. Reassessing disease characteristics at progression may offer important insights, especially in guiding clinical trial participation and personalized treatment. Further studies are needed to deepen our understanding of the dynamic nature of metastatic panNET.

## AUTHOR CONTRIBUTIONS

**Kazhan Mollazadegan:** Conceptualization; investigation; funding acquisition; supervision; formal analysis; validation; writing – review and editing; writing – original draft; project administration; resources; visualization; data curation. **Johan Botling:** Conceptualization; writing – review and editing; resources; validation; investigation; visualization; writing – original draft. **Britt Skogseid:** Funding acquisition; resources; writing – review and editing. **Barbro Eriksson:** Resources; funding acquisition. **Lovisa Falkman:** Writing – review and editing. **Liang Zhang:** Writing – review and editing. **Ieva Lase:** Writing – review and editing. **Staffan Welin:** Resources; writing – review and editing; funding acquisition. **Anders Sundin:** Conceptualization; writing – original draft; writing – review and editing; visualization; investigation; resources; validation. **Joakim Crona:** Conceptualization; investigation; funding acquisition; writing – original draft; visualization; validation; writing – review and editing; project administration; supervision; resources.

## FUNDING INFORMATION

This study was funded by a European Neuroendocrine Tumor Society young investigator grant (JC) as well as grants from Bengt Ihres fond (JC), Lions cancerforskningsfond (JC), Nordic Neuroendocrine Tumor Group (JC), Torsten och Ragnar Söderbergs Stiftelse (JC), Åke Wibergs Stiftelse (JC), Lennart Glans Stiftelse (JC), Region Uppsala ALF (JC, KM) and Cancerfonden (JC). The funders had no role in the design, data collection, data analysis, and reporting of this study.

## CONFLICT OF INTEREST STATEMENT

All authors declare no conflict of interest.

## PEER REVIEW

The peer review history for this article is available at https://www.webofscience.com/api/gateway/wos/peer‐review/10.1111/jne.70040.

## ETHICS STATEMENT

The authors of this article affirm their commitment to the highest ethical standards in the conduct and reporting of research. Before initiating the study, approval was obtained from the Ethical Review Board in Uppsala (reference ID: 2016/277). Participants were fully informed about the nature and purpose of the research, and their voluntary participation was ensured. Written informed consent was obtained from all patients. Identifying information was removed or anonymized to safeguard the privacy of individuals.

## Supporting information


**Table S1.** Outcomes following the first re‐characterization. Before study inclusion: biopsy or surgery performed at primary diagnosis; n/a: not applicable, no information available, data missing; +/−: positive/negative; FDG:[^18^F]fluorodeoxyglucose; significant changes: defined as (i) the occurrence of a new hormonal syndrome or relevant hormone secretion, (ii) changes in PET‐tracer uptake pattern (loss or gain of ^18^F‐FDG‐ or ^68^Ga‐DOTATOC‐positive tumor lesions), (iii) an increase in Ki‐67 index resulting in a transition from a low grade to a higher grade (G) pancreatic neuroendocrine tumor (panNET), or low G2 to high G2, leading to changes in treatment strategy according to ENETS guidelines (1).
**Table S2.** NETPET‐score following the first re‐characterization. n/a: not applicable, data missing; +/−: positive/negative; SSTR: somatostatin receptor; FDG:[^18^F]fluorodeoxyglucose; NETPET score: P0: SSTR and FDG negative, P1: SSTR positive, FDG negative, P2a: FDG<SSTR (1–2 lesions), P2b: FDG<SSTR (3 or more lesions), P3a: FDG = SSTR (1–2 lesions), P3b: FDG = SSTR (3 or more lesions), P4a: FDG>SSTR (1–2 lesions), P4b FDG>SSTR (3 or more lesions), P5: FDG positive, SSTR negative.
**Table S3.** Overall survival – following the first re‐characterization. Median survival was 20 months. *Died shortly after 1st re‐characterization; n/a: not applicable, no information available, PET scan or biopsy not performed, still alive at the end of the study; SSTR: somatostatin receptor; FDG:[^18^F]fluorodeoxyglucose; NETPET‐score: P0: SSTR and FDG negative, P1: SSTR positive, FDG negative, P2a: FDG<SSTR (1–2 lesions), P2b: FDG<SSTR (3 or more lesions), P3a: FDG = SSTR (1–2 lesions), P3b: FDG = SSTR (3 or more lesions), P4a: FDG>SSTR (1–2 lesions), P4b FDG>SSTR (3 or more lesions), P5: FDG positive, SSTR negative.
**Table S4.** Outcomes after the second re‐characterization. n/a: not applicable, no information available, PET scan or biopsy not performed; +/−: positive/negative; FDG:[^18^F]fluorodeoxyglucose; significant changes: defined as (i) the occurrence of a new hormonal syndrome or relevant hormone secretion, (ii) changes in PET‐tracer uptake pattern (loss or gain of ^18^F‐FDG‐ or ^68^Ga‐DOTATOC‐positive tumor lesions), (iii) an increase in Ki‐67 index resulting in a transition from a low grade to a higher grade (G) pancreatic neuroendocrine tumor (panNET), or low G2 to high G2 panNET, leading to changes in treatment strategy according to ENETs guidelines (2,3). *Information about therapy change has also been added for patients not undergoing re‐characterization according to the study protocol, **Atypical lever resection of single metastatic progressive lesion, but no change of systemic therapy.
**Table S5.** NETPET‐grade following the second re‐characterization. n/a: not applicable, PET scan not performed; +/−: positive/negative; SSTR: somatostatin receptor; FDG:[^18^F]fluorodeoxyglucose; NETPET‐score: P0: SSTR and FDG negative, P1: SSTR positive, FDG negative, P2a: FDG<SSTR (1–2 lesions), P2b: FDG<SSTR (3 or more lesions), P3a: FDG = SSTR (1–2 lesions), P3b: FDG = SSTR (3 or more lesions), P4a: FDG>SSTR (1–2 lesions), P4b FDG>SSTR (3 or more lesions), P5: FDG positive, SSTR negative.
**Table S6.** Systemic therapy given before inclusion and after the first and second re‐characterization. STZ: streptozotocin; 5FU: 5‐flourouracil; PRRT: peptide receptor radionuclide therapy; TMZ: temozolomide; CAPTEM: capecitabine/temozomolide; mTORi: mTOR inhibitor/afinitor; SSA: somatostatin analogue; CE: carboplatin/etoposide; INF‐α: Interferon alfa; IRE: irreversible electroporation. *Information about therapy change has been added also for patients not undergoing re‐characterization according to the study protocol.

## Data Availability

All data generated or analyzed during this study are included in this article and its Table S1 files. Further inquiries can be directed to the corresponding author.
