# Supplementary material for: The impact of re‐characterizing metastatic pancreatic neuroendocrine tumors: A prospective study
Source: J Neuroendocrinol. 2025 May 5;37(8):e70040. doi: 10.1111/jne.70040 (PMC12358203; doi:10.1111/jne.70040)
Supplement: Supplementary file 1 — Table S1. Outcomes following the first re‐characterization. Before study inclusion: biopsy or surgery performed at primary diagnosis; n/a: not applicable, no information available, data missing; +/−: positive/negative; FDG:[18F]fluorodeoxyglucose; significant changes: defined as (i) the occurrence of a new hormonal syndrome or relevant hormone secretion, (ii) changes in PET‐tracer uptake pattern (loss or gain of 18F‐FDG‐ or 68Ga‐DOTATOC‐positive tumor lesions), (iii) an increase in Ki‐67 index resulting in a transition from a low grade to a higher grade (G) pancreatic neuroendocrine tumor (panNET), or low G2 to high G2, leading to changes in treatment strategy according to ENETS guidelines (1). Table S2. NETPET‐score following the first re‐characterization. n/a: not applicable, data missing; +/−: positive/negative; SSTR: somatostatin receptor; FDG:[18F]fluorodeoxyglucose; NETPET score: P0: SSTR and FDG negative, P1: SSTR positive, FDG negative, P2a: FDG<SSTR (1–2 lesions), P2b: FDGSSTR (1–2 lesions), P4b FDG>SSTR (3 or more lesions), P5: FDG positive, SSTR negative. Table S3. Overall survival – following the first re‐characterization. Median survival was 20 months. *Died shortly after 1st re‐characterization; n/a: not applicable, no information available, PET scan or biopsy not performed, still alive at the end of the study; SSTR: somatostatin receptor; FDG:[18F]fluorodeoxyglucose; NETPET‐score: P0: SSTR and FDG negative, P1: SSTR positive, FDG negative, P2a: FDG<SSTR (1–2 lesions), P2b: FDGSSTR (1–2 lesions), P4b FDG>SSTR (3 or more lesions), P5: FDG positive, SSTR negative. Table S4. Outcomes after the second re‐characterization. n/a: not applicable, no information available, PET scan or biopsy not performed; +/−: positive/negative; FDG:[18F]fluorodeoxyglucose [file JNE-37-e70040-s001.docx]

| Patient No. | Ki-67 (%) before study inclusion | Ki-67 (%) | FDG-PET status (+/-) | New FDG-avid lesions (+/-) | Increased uptake of known FDG-avid lesions (+/-) | ≥2x baseline  Ki-67 | New hormone secretion (+/-) | New hormonal syndrome (+/-) | Hormone | Significant change (+/-) | Therapy change  (Yes +/No -) | Time from primary diagnosis to 1^st^ re-characterization (months) |
| --- | --- | --- | --- | --- | --- | --- | --- | --- | --- | --- | --- | --- |
| 1 | 7 | n/a | - | n/a | n/a | n/a | + | - | Calcitonin | + | + | 97 |
| 2 | 15 | 16 | + | n/a | n/a | - | n/a | n/a |  | - | + | n/a |
| 3 | 10 | n/a | - | n/a | n/a | - | n/a | n/a |  | n/a | + | 30 |
| 4 | 31 | 20 | + | n/a | n/a | - | n/a | n/a |  | - | n/a | 14 |
| 5 | 5 | 9 | + | n/a | n/a | - | n/a | n/a |  | - | - | 149 |
| 6 | 7 | n/a | + | n/a | n/a | - | - | n/a |  | - | n/a | 54 |
| 7 | 12 | 30 | + | n/a | n/a | + | - | - |  | + | n/a | 24 |
| 8 | 10 | 14 | + | n/a | n/a | - | + | - | PP | + | - | 2 |
| 9 | 12 | 60 | + | n/a | n/a | + | + | - | VIP | + | - | 15 |
| 10 | 3 | 10 | - | n/a | n/a | + | - | - |  | + | - | 160 |
| 11 | 12 | 16 | + | n/a | n/a | - | - | - |  | - | - | 4 |
| 12 | 4 | 12 | + | + | + | + | - | - |  | + | - | 181 |
| 13 | 2 | n/a | - | n/a | n/a | n/a | n/a | n/a |  | n/a | + | 63 |
| 14 | 4 | 33 | + | n/a | n/a | + | + | - | Gastrin | + | + | 24 |
| 15 | 14 | 70 | + | n/a | n/a | + | - | - |  | + | + | 45 |
| 16 | 8 | 6 | + | n/a | n/a | - | - | - |  | - | - | 100 |
| 17 | 11 | 21 | + | + | n/a | + | - | - |  | + | + | 35 |
| 18 | 18 | 40 | + | + | - | + | n/a | n/a |  | + | + | 9 |
| 19 | 25 | n/a | + | n/a | + | - | - | - |  | + | - | 17 |
| 20 | 7 | 21 | + | n/a | n/a | + | - | - |  | + | + | 58 |
| 21 | 3 | 40 | + | n/a | n/a | + | - | - |  | + | + | 48 |
| Total sum |  |  | 17 | 3 | 2 | 10 | 4 | 0 |  | 13 | 10 |  |
| Median |  |  |  |  |  |  |  |  |  |  |  | 35 |

**Supplementary Table 1.** Outcomes following the first re-characterization.

**Supplementary Table 2.** NETPET-score following the first re-characterization

| Patient No. | NETPET-score | SSTR-scan status (+/-) | FDG-scan status (+/-) | Description of target lesions, most FDG avid relative to SSTR | Secondary Characteristics |
| --- | --- | --- | --- | --- | --- |
| **4** | P2b | + | + |  |  |
| **8** | P2b | + | + |  |  |
| **9** | P2b | + | + |  |  |
| **10** | P1 | + | - | Only SSTR+ |  |
| **11** | P2b | + | + |  |  |
| **12** | P2b | + | + | only 3 FDG+, majority SSTR+ |  |
| **13** | P1 | + | - |  |  |
| **14** | P2b | + | + | Only 3 FDG+ lesions, majority SSTR+ |  |
| **15** | P2b | + | + |  |  |
| **16** | P2b | + | + | Majority SSTR+ |  |
| **17** | P2b | + | + | Majority SSTR+ |  |
| **18** | P2b | + | + | 4 FDG+, majority SSTR+ | 5 lesions on CT, SSTR and FDG-PET negative. |
| **19** | P3b | + | + | n/a | with additional >3 lesions FDG<SSTR |
| **20** | P2b | + | + | n/a |  |
| **21** | P2b | + | + | n/a | with additional 2 lesions FDG<SSTR |

**Supplementary table 3.** Overall survival – following the first re-characterization

| Patient No. | Survival status and follow-up time, months | SSTR-PET performed  (+/-) | FDG-PET performed (+/-) | Ki-67% | NETPET-score |
| --- | --- | --- | --- | --- | --- |
| 1 | Diseased, 30 | n/a | + | n/a | n/a |
| 2 | Diseased, 17 | n/a | + | 16 | n/a |
| 3 | Diseased, 35 | n/a | + | n/a | n/a |
| 4 | Diseased, 3* | + | + | 20 | P2b |
| 5 | Diseased, 15 | n/a | + | 9 | n/a |
| 6 | Diseased, 14 | n/a | + | n/a | n/a |
| 7 | Diseased, 3* | n/a | + | 30 | n/a |
| 8 | Diseased, 36 | + | + | 14 | P2b |
| 9 | Alive, 29 | + | + | 60 | P2b |
| 10 | Alive, 29 | + | + | 10 | P1 |
| 11 | Alive, 28 | + | + | 16 | P2b |
| 12 | Alive, 33 | + | + | 12 | P2b |
| 13 | Alive, 65 | + | + | n/a | P1 |
| 14 | Diseased, 10 | + | + | 33 | P2b |
| 15 | Diseased, 5* | + | + | 70 | P2b |
| 16 | Alive, 27 | + | + | 6 | P2b |
| 17 | Diseased, 18 | + | + | 21 | P2b |
| 18 | Diseased, 11 | + | + | 40 | P2b |
| 19 | Diseased, 2* | + | + | n/a | P3b |
| 20 | Alive, 22 | + | + | 21 | P2b |
| 21 | Diseased, 20 | + | + | 40 | P2b |

| Patient No. | 1^st^ re-characterization Ki-67 (%) | Ki-67 (%) | FDG-PET status (+/-) | New FDG-avid lesions (+/-) | ≥2x Ki-67 1^st^ re-characterization | New hormone secretion (+/-) | Hormonal syndrome (+/-) | Hormone | Significant change (+/-) | Therapy change*  Yes +, No - |
| --- | --- | --- | --- | --- | --- | --- | --- | --- | --- | --- |
| 1 | n/a | n/a | n/a | n/a | n/a | n/a | n/a |  | n/a | + |
| 2 | 16 | n/a | n/a | n/a | n/a | n/a | n/a |  | n/a | + |
| 3 | n/a | 10 | n/a | n/a | - | - | - |  | - | + |
| 4 | 20 | n/a | n/a | n/a | n/a | n/a | n/a |  | n/a | n/a |
| 5 | 9 | n/a | n/a | n/a | n/a | + | - | VIP | + | n/a |
| 6 | n/a | n/a | n/a | n/a | n/a | - | - |  | - | n/a |
| 7 | 30 | n/a | n/a | n/a | n/a | - | - |  | - | n/a |
| 8 | 14 | 30 | + | + | + | + | - | Gastrin | + | + |
| 9 | 60 | n/a | n/a |  |  | n/a | n/a |  | n/a | n/a |
| 10 | 10 | n/a | n/a |  |  | n/a | n/a |  | n/a | n/a |
| 11 | 16 | n/a | n/a |  |  | n/a | n/a |  | n/a | n/a |
| 12 | 12 | 14 | + | - | - | n/a | n/a |  | - | + |
| 13 | n/a | n/a | - | - | n/a | n/a | n/a |  | n/a | + |
| 14 | 33 | n/a | n/a |  |  | n/a | n/a |  | n/a | n/a |
| 15 | 70 | n/a | n/a |  |  | n/a | n/a |  | n/a |  |
| 16** | 6 | 12 | n/a |  | + | n/a | n/a |  | + | n/a |
| 17 | 21 | 33 | + | - | - | n/a | n/a |  | - | + |
| 18 | 40 | n/a | n/a |  |  | n/a | n/a |  | n/a | + |
| 19 | n/a | n/a | n/a |  |  | n/a | n/a |  | n/a | n/a |
| 20 | 21 | n/a | n/a |  |  | n/a | n/a |  | n/a | n/a |
| 21 | 40 | n/a | n/a |  |  | n/a | n/a |  | n/a | n/a |
| Total sum |  |  |  | 1 | 2 | 2 |  |  | 3 | 8 |

**Supplementary Table 4.** Outcomes after the second re-characterization

**Supplementary Table 5.** NETPET-grade following the second re-characterization

| Patient No. | NETPET-score | SSTR-scan status (+/-) | FDG-scan status (+/-) | Description of target lesions most FDG avid relative to SSTR | Secondary Characteristics |
| --- | --- | --- | --- | --- | --- |
| 8 | n/a | n/a | + | n/a | n/a |
| 9 | n/a | + | n/a | n/a | n/a |
| 12 | P2b | + | + | n/a | Peritoneal carcinomatosis |
| 13 | P1 | + | - |  | New SSTR+ lesions. |

**Supplementary table 6 –** Systemic therapy given before inclusion and after the first and second re-characterization

|  | **Before inclusion** | | | |  | **1^st^ re-characterization** | |  | **2^nd^ re-characterization** |
| --- | --- | --- | --- | --- | --- | --- | --- | --- | --- |
| **Patient No.** | **1^st^ line** | **2^nd^ line** | **3^rd^ line** | **4^th^ line** |  | **1^st^ line** | **2^nd^ line** |  | **1^st^ line*** |
| **1** | STZ/5FU | PRRT | SSA | SSA + PRRT x 3 |  | SSA + CAPTEM |  |  | SSA + mTORi |
| **2** | SSA + CAPTEM | SSA + PRRT x 5 | SSA + PRRTx 3 |  |  | SSA + STZ/5FU x 1 |  |  | SSA + mTORi |
| **3** | STZ/5FU | CAPTEM | PRRT x 5 |  |  | SSA |  |  | SSA + PRRT x 3 |
| **4** | CAPTEM | CE |  |  |  |  |  |  |  |
| **5** | TMZ | SSA+ TMZ |  |  |  | SSA + TMZ |  |  |  |
| **6** | SSA + STZ/5FU | SSA + CAPTEM | SSA + PRRT x 4 |  |  |  |  |  |  |
| **7** | STZ/5FU | PRRT x 4 |  |  |  |  |  |  |  |
| **8** |  |  |  |  |  | STZ/5FU | SSA |  | SSA + TMZ |
| **9** |  |  |  |  |  | CAPTEM |  |  |  |
| **10** | SSA+ INF-α | SSA + PRRT x 6 |  |  |  | SSA + PRRT x 3 |  |  | NA |
| **11** |  |  |  |  |  | CAPTEM | PRRT x4 |  |  |
| **12** | SSA+ STZ/5FU | SSA + PRRT x 6 | SSA + mTORi | SSA + PRRT x 3 |  | SSA + PRRT x 3 |  |  | SSA + CAPTEM |
| **13** | STZ/5FU |  |  |  |  | IRE |  |  | PRRT x 5 |
| **14** | SSA |  |  |  |  | SSA+ PRRT x2 (clinical detoriation) |  |  |  |
| **15** | SSA + STZ/5FU | SSA + TMZ |  |  |  | SSA + PRRT x 2 |  |  |  |
| **16** | SSA + STZ/5FU | SSA + TMZ | SSA + TMZ/5FU |  |  | SSA + TMZ/5FU | SSA + Atypical liver resection |  |  |
| **17** | SSA + STZ/5FU |  |  |  |  | SSA + PRRT x 4 |  |  | SSA + CAPTEM |
| **18** | CAPTEM |  |  |  |  | CE |  |  | PRRT |
| **19** | mTORi | TMZ + mTORi |  |  |  | TMZ |  |  |  |
| **20** | SSA + STZ/5FU |  |  |  |  | SSA + PRRT x 4 |  |  |  |
| **21** | SSA + CAPTEM | SSA + STZ/5FU | SSA + PRRT x 4 | SSA + mTORi |  | SSA + CAPTEM |  |  |  |
